# Exploring the role of preprocessing combinations in hyperspectral imaging for deep learning colorectal cancer detection

**DOI:** 10.1038/s41598-025-20735-x

**Published:** 2025-09-23

**Authors:** Mariia Tkachenko, Benjamin Huber, Serhii Hamotskyi, Boris Jansen-Winkeln, Ines Gockel, Thomas Neumuth, Hannes Köhler, Marianne Maktabi

**Affiliations:** 1 Center for scalable data analytics and artificial intelligence (ScaDS.AI) Dresden/Leipzig, Leipzig, Germany; 2 Innovation Center Computer-Assisted Surgery (ICCAS), University of Leipzig, Leipzig, 04103 Germany; 3 Anhalt University of Applied Sciences, Köthen, 06366 Germany; 4 Department of Visceral, Transplant, Thoracic and Vascular Surgery, University Hospital of Leipzig, Leipzig, 04103 Germany; 5 Department of Gastrointestinal Surgery, IRCCS San Raffaele Scientific Institute and San Raffaele Vita-Salute University, Milan, 20132 Italy

**Keywords:** Preprocessing, Cancer classification, Colorectal cancer, Hyperspectral imaging, Machine learning, Convolutional networks, Oncology, Cancer, Cancer imaging, Cancer, Cancer imaging

## Abstract

This study compares various preprocessing techniques for hyperspectral deep learning–based cancer diagnostics. The study considers different spectrum scaling and noise reduction options across spatial and spectral axes of hyperspectral datacubes, as well varying levels of blood and light reflections removal. We also examine how the size of the patches extracted from the hyperspectral data affects the models’ performance. We additionally explore various strategies to mitigate our dataset’s imbalance (where cancerous tissues are underrepresented). Our results indicate that. Scaling: Standardization significantly improves both sensitivity and specificity compared to Normalization. Larger input patch sizes enhance performance by capturing more spatial context. Noise reduction unexpectedly degrades performance. Blood filtering is more effective than filtering reflected light pixels, although neither approach produces significant results. By carefully maintaining consistent testing conditions, we ensure a fair comparison across preprocessing methods and reproducibility. Our findings highlight the necessity of careful preprocessing selection to maximize deep learning performance in medical imaging applications.

**Supplementary Information:**

The online version contains supplementary material available at 10.1038/s41598-025-20735-x.

## Introduction

Cancer is one of the leading causes of death worldwide^1^. Colorectal cancer — third by incidence and second by mortality^2^ — accounts for a significant portion of cancer-related morbidity. Techniques usable during preoperative^3^, intraoperative^4^, or postoperative stages (e.g., to detect cancer regions and ensure tumor-free resection margins^5^ can aid in cancer diagnosis and improve outcomes.

Hyperspectral imaging (HSI) combines imaging and spectroscopy, creating image data containing both spatial information and the spectrum for each pixel, usually extending beyond the wavelengths seen by humans. HSI has shown potential in discriminating between tissue structures by analyzing their spectrum, thereby distinguishing healthy and pathological (e.g., cancerous) tissues in a contactless, radiation-free and non-invasive manner, and has seen increasing adoption in the medical field^4^.

HSI has been used for cancer detection in humans, for skin^6,7^, breast^8^, head and neck^9^, brain^10,11^, oral^12^, gastric^13^ cancer, as well as colorectal cancer^14,15^, and various others.

Different approaches exist for extracting this information from hypercubes: both conventional Machine Learning (ML) models, which have a stronger reliance on domain-specific knowledge to extract data, and Deep Learning (DL) ones, which can discover structures in high-dimensional data with very little engineering by hand^16^ and have obtained better classification performance than traditional approaches^17^. DL models used for HSI cancer detection have been primarily (but not exclusively) convolutional neural networks (CNNs)^18–21^, including 3D-CNNs^14,22^. Other approaches have been used with success as well, for instance semi-supervised Graph Convolutional Networks that leverage the use of unlabeled pixels^23^.

Cancer detection with HSI and DL is challenging already at the dataset acquisition stage: HSI and ground truth labeling require devices and medical expertise, resulting in smaller datasets than usually found in non-medical domains (e.g., in the order of tens, not thousands, of images).

Datasets can also include class imbalance (e.g., more cancerous pixels than healthy ones), light or blood reflections, and variations in tissue structure, all presenting challenges in developing accurate and robust models. Blood poses a problem due to its strong absorption and scattering properties, which can distort spectral signatures and reduce the contrast between cancerous and non-cancerous tissues — a previously neglected dimension that our work studies in detail.

Previous studies have explored the impact of pre- and post-processing steps on the supervised classification of tissues in hyperspectral images, highlighting the importance of optimized data processing techniques^8,22,24^.

This study builds upon these findings by addressing the challenge of optimizing the preprocessing of HSI data with the use of 3D-CNNs, contributing in two separate but interrelated ways: (1) The comprehensive exploration of the effects of various preprocessing approaches (including noise reduction by smoothing and blood filtering, the latter especially being overlooked in literature), and (2) A pipeline enabling systematic testing of preprocessing combinations under the conditions where testing all combinations thoroughly is prohibitively difficult, with an emphasis on validity, statistically sound methods, and exclusion of confounding variables.

Various combinations of preprocessing are explored, including scaling, smoothing, filtering blood and light reflection pixels, and different approaches to address dataset imbalance: class weighting and sample weighting (to increase model sensitivity to smaller annotated areas).

Evaluating each combination of parameters on the entire dataset would be unfeasibly time and resource intensive. Combinations were tested on a smaller, statistically representative subset of the dataset. Reproducibility and reliability are ensured by a careful exclusion of confounding variables and fixing possible points of randomness, by controlling weight initialization/dropout/random seeds, and ensuring consistent ordering. This ensures robust results and at the same time can serve as a blueprint for other experiments under similar constraints.

## Data

**Fig. 1:**
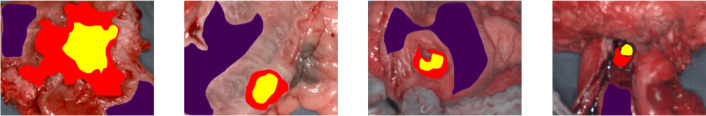
Examples of masks with ground truth labels: purple indicates non-malignant tissue, yellow represents cancerous tissue, and red denotes the margin of cancerous tissue.

The dataset includes HS datacubes from 56 patients, annotated by expert pathologists and surgeons based on histopathological slide comparisons. Each patient’s record contains an HS cube and an associated image (ground truth mask) that displays ground truth labels, shown in Fig. 1. In this study, we focus on binary classification of tissues, specifically identifying non-malignant (purple) and cancerous (yellow) tissues. It is important to note the significant imbalance in the dataset, which contains approximately 10 non-malignant to 1 cancerous pixel, in total 477,670 cancerous pixels and 4,537,769 non-malignant pixels.

The HS image data of resected colorectal tissue was captured within the first five minutes post-resection, using the push-broom TIVITA^®^ Tissue device from Diaspective Vision GmbH, Germany, which was positioned 50 cm away from the tissue and all side lighting was excluded. Each image required approximately ten seconds to capture. The generated datacubes have dimensions of 480 by 640 pixels with 100 spectral bands, spanning a wavelength range from 500 to 1000 nm at 5 nm intervals. We excluded the wavelengths between 500 and 540 nm due to excessive noise, resulting in the use of 92 spectral bands corresponding to the 540–1000 nm range.

## Methods

The study was approved by the local ethics committee of the medical Faculty of the University of Leipzig (026/18-ek), the study was also registered at Clinicaltrials.gov (NCT04230603). Written informed consent, including consent for publication, was obtained from all the patients. All methods were performed in accordance with the Declaration of Helsinki.

### Ensuring compatibility between combinations

A machine learning pipeline can be viewed as comprising the following independent components: Data, Preprocessing, Model, and Cross-validation.

These components are independent in the sense that, to compare different variations of one component, the remaining components must be held constant. In this study, to isolate the effect of preprocessing, all other factors including data order, model state and cross-validation were held constant. In Table 1 we outline the checklist used to ensure consistency across each component. These prerequisites ensured that the only variable altered was the preprocessing combination. Consequently, if preprocessing A outperformed preprocessing B, we could be confident that the observed differences were solely due to the preprocessing methods, with no confounding variables influencing the results.

**Table 1:** Checklist for fixing randomness.

Data	Same data in the same order	It is essential to ensure that the model (neural network) receives the same data in the same order, with preprocessing being the only variable component.
	Representative subset	Evaluating all combinations on the full dataset would be impractical because the time required for testing even a single combination would take 5–6 days. However, instead of selecting a random subset of the dataset, we aimed to choose a subset that is *representative* of the entire dataset’s characteristics. We developed an algorithm for this purpose, described in Sect. 3.1.1.
Model	Fixed models	For all preprocessing combinations, we used the same 3D-CNN model, described in Sect. 3.3.1.
	Weights and biases initialization	We initialized weights and biases always to the same values to ensure that the starting point of training is the same.
	Ensure that randomness inside layers like Dropout is turned off	Some layers use randomness, e.g., Dropout that randomly drops connections to avoid overfitting. It is important to set the same seed to such layers to make their behavior reproducible.
	Set a seed to Tensorflow, Python and other libraries	Set a seed to Tensorflow, Python and other libraries that may contain randomization to eliminate effects of other possible random operations.
Cross-validation	Mapping between test patients and validation patients	We used Leave-One-Out-Cross-Validation (Sect. 3.3.1.), effectively 56-fold cross-validation (one-fold for each patient). In each fold, we excluded 1 patient as test set, and 3 patients as validation set, all other patients were in the train set. To ensure fair comparison between combinations, a mapping between test patients and validation patients was created that was used for all combinations. In other words, each time Patient X was excluded as test set in any preprocessing combination, the same corresponding 3 patients were chosen for the validation set.

#### Sampling a small but representative dataset

First, we needed to create a small representative subset as follows:

1. Randomly select 1% of data from each patient and each class.
2. Apply Kolmogorov–Smirnov test (K-S test, α = 0.05) for every wavelength to confirm that the empirical distribution in the reduced subset does not differ significantly from the corresponding distribution in the full dataset, thereby establishing that the subset is distributionally representative.
3. Repeat until distributions of all wavelengths are representative to the whole dataset. In most cases, less than 5 repetitions were needed.

Afterwards, for each preprocessing combination, corresponding preprocessing was performed on the selected small representative prototype. This way we ensured that the model received the same data in the same order, only the preprocessing was different.

### Preprocessing

#### Pipeline

The preprocessing pipeline comprised the following sequential steps:

1. Reading Hyperspectral Datacube and Ground Truth Mask. The initial step involved importing the hyperspectral data along with the corresponding ground truth mask, which provided reference labels.
2. Scaling. This step scaled the data values, using Normalization or Standardization methods to ensure uniformity and better convergence of neural networks.
3. Smoothing. Noise reduction was achieved through smoothing techniques.
4. Filtering.Filtering processes like blood and light reflection filtering were applied to remove possibly harmful artifacts.
5. Finally, for each annotated pixel we extracted 3D patches, which became the input samples for neural networks. Each patch was generated from a labeled pixel along with its neighboring pixels. Patch sizes in this work were 3 and 5, so the input shapes of the samples were (3, 3, 92) and (5, 5, 92).

#### Scaling

**Fig. 2:**
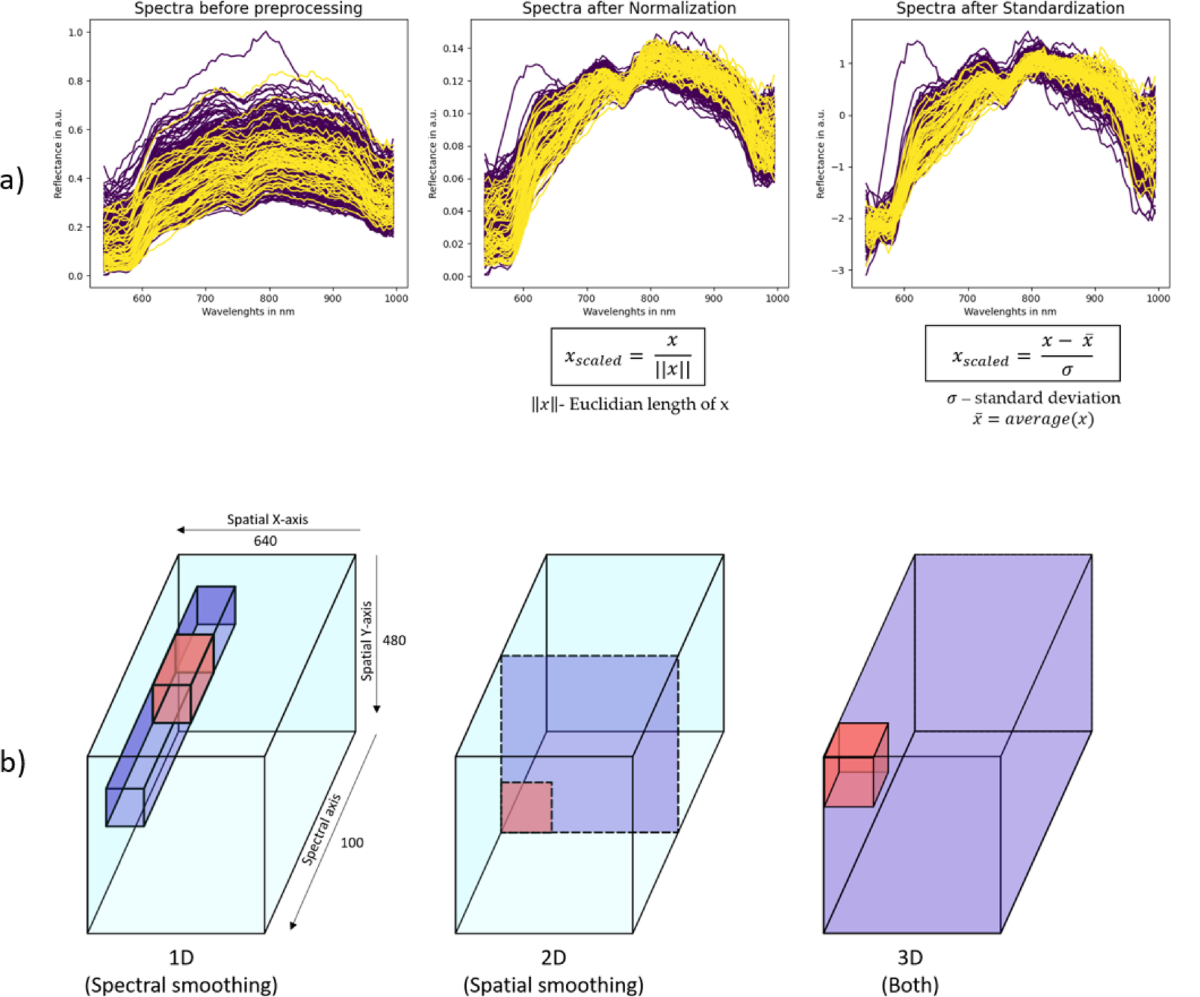
Preprocessing techniques. (**a**) Spectra examples before (left) and after scaling (Normalization in the middle, Standardization on the right). Note the differing scales on the y-axes. (**b**) Three modes of smoothing: 1D, 2D and 3D, where the purple areas represent the region being subjected to the smoothing process at once for the respective mode (1D, 2D or 3D), while the red areas highlight the window which would be applied pixel by pixel sequentially across the purple area. The datacubes’ axis proportions are drawn not to scale for clarity.

Scaling is important not only for bringing all features into a consistent range, which enhances convergence, but also for eliminating biases, such as these stemming from unique spectral characteristics of each patient. To address this, we employed feature scaling techniques. Based on previous research^22^, we selected two algorithms: Normalization (scaling to unit length) and Standardization (Z-score). Figure 2a presents the formulas for these scaling methods and illustrates how each scaling affects the spectra.

#### Smoothing

To reduce noise, we tried 1D (spectral), 2D (spatial), and 3D (both) smoothing on our datacubes as part of preprocessing pipelines. Figure 2b illustrates these different approaches within a hyperspectral cube, where the purple areas represent the region being subjected to the smoothing process at the same time for the respective mode (1D, 2D or 3D), while the red areas highlight the window applied pixel by pixel across the purple area. 1D smoothing is applied along the spectral dimension. 2D smoothing is applied spatially to each spectral band. 3D smoothing involves the simultaneous application of filters across both spatial and spectral dimensions.

Three filters were chosen for smoothing: Median Filter (MF), Gaussian Filter (GF) and Savitsky-Golay Filter (SGF)^25^, the use of which led to a significant improvement in^14^. MF replaces each pixel value with the median of neighboring values within a specified window size. GF, in contrast, applies a Gaussian function with a specific *sigma* value to weigh the neighboring spectral values. SGF was applied only with 1D smoothing.

The sigmas used for GF and window sizes used for MF for each dimension are described in Sect. 3.2.6. These values were selected based on a comparative analysis of spectra visualizations before and after the application of smoothing, ensuring the chosen parameters effectively modified the spectra while preserving essential structural details. SGF was applied with a window size of 9 and a polynomial order of 2, as these parameters yielded the best results in the study by Collins et al.^14^.

#### Filtering

**Fig. 3:**
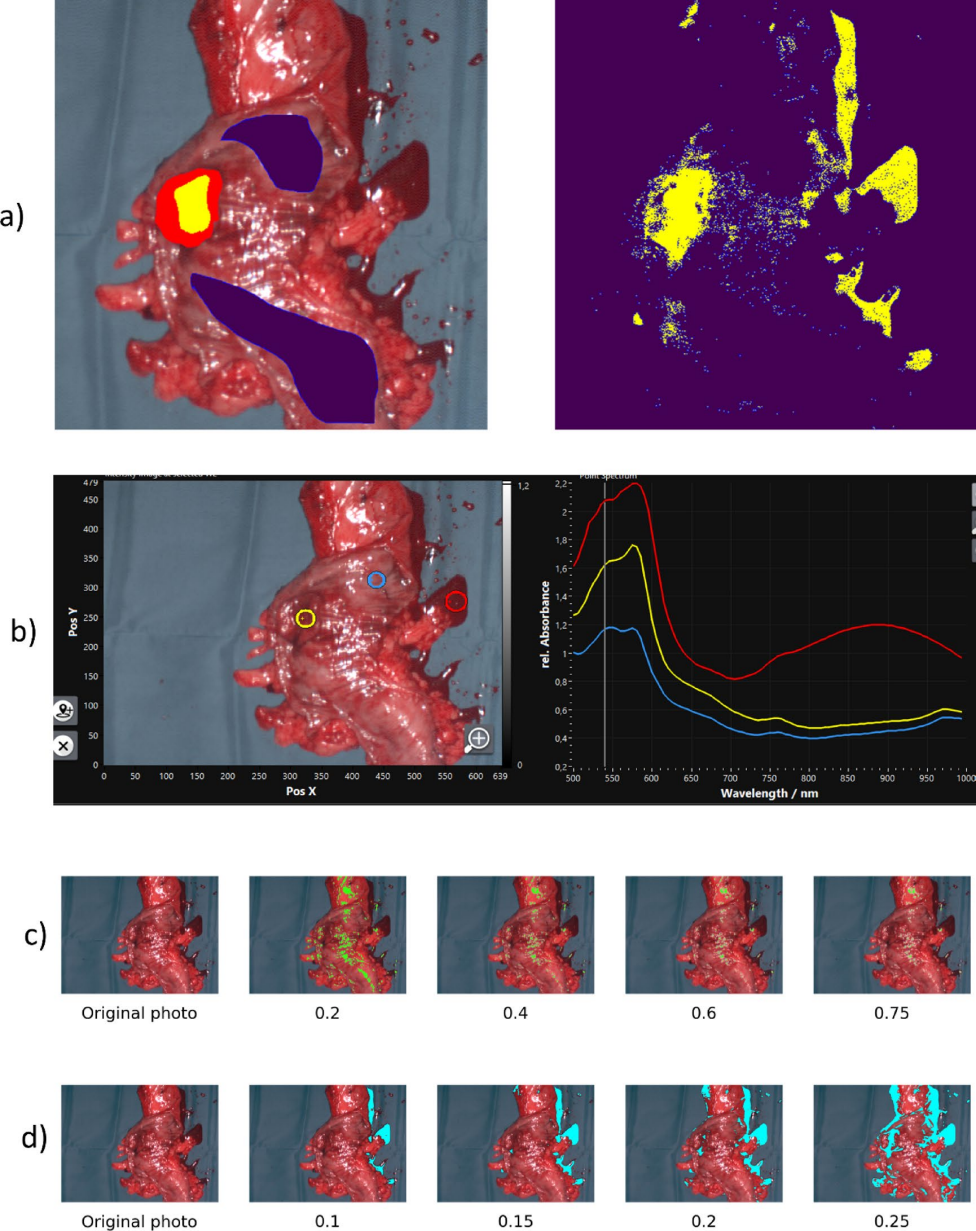
Filtering (**a**) Left: ground truth, note the blood. Right: prediction map that shows how blood is classified as cancer. Yellow shows cancerous tissues and violet non-malignant ones. (**b**) Screenshot from TIVITA Suite (Diaspective Vision GmbH, Am Salzhaff-Pepelow). The right picture shows spectra from the circled areas of the left picture, in the same colors. Blue is healthy tissue, yellow — cancerous tissue, red is blood. Note how the blood spectrum is much more similar to the cancerous spectrum than to the non-malignant. (**c**) Regions detected as reflected light (green) at different “light thresholds”. (**d**) Regions detected as blood at different “blood thresholds” are shown in cyan. Summary: blood spectra are more similar to cancer than to healthy tissue, explaining the high false‑positive rate and motivating blood‑pixel filtering.

During our research, it was shown that our models tended to misclassify pixels with reflected light, and to a higher extent — pixels with blood(Fig. 3a). The likely cause is that the blood spectra are similar to cancerous spectra, as shown in Fig. 3b.

Considering the above observations, we decided to exclude blood and light reflection pixels using the method for background extraction described in^26^ (Sect. 2.4). The background detection algorithm works by calculating three metrics — A, B, and C — from the hyperspectral image data. Metric A is the mean reflectance value over the wavelength range of 500 to 1000 nm, and metric B is the mean reflectance value within the 510 to 570 nm range. Metric C is a logarithmic function that compares the reflectance within the 650 to 710 nm range to a constant value. The pixel is classified as background, if A is less than 0.1, B is larger than 0.7, and C is negative. For clarity, in this work thresholds for these same *A* and *B* will be referred to as “light threshold” and “blood threshold”, respectively. In^26^, the light threshold was 0.1, and the blood threshold was set to 0.7. In this work, we tested different values for both thresholds to investigate effects on training outcomes. Figure 3c and d shows how many pixels were identified as light and blood for different threshold values.

#### Sample and class weights

With 477,670 cancerous and 4,537,769 non-malignant pixels, our dataset is imbalanced. To address this issue, we used class weights during training. Class weights are defined with the following formula:

$$class\_weigh{t_{class~C}}=\frac{{total~number~of~pixels}}{{number~of~pixels~of~class~C}}$$

Class weights are applied to the loss function. And in this case, for example, if cancerous tissue is misclassified, the loss function will be multiplied by class weight for cancerous tissue (~ 10 in our case). Hence, the ML model learns to pay more attention to the less represented classes.

In Fig. 1, it can be seen that the cancer area of the far-right patient is significantly bigger than the cancer area of the far-left patient. During previous research, it has been established that ML models perform better on patients who have bigger cancer areas, which seems reasonable, because patients with bigger cancer are more represented. But it is important to pay attention to the patients with smaller cancer areas as well, because each patient could contribute to a better generalization of the model. Therefore, we included sample weights in our tests. Sample weights are defined as:

$$sample\_weigh{t_{class~C,~patient~P}}\frac{{total~number~of~pixels}}{{number~of~pixels~of~class~C~of~patient~P}}$$

If sample weights were used, class weights were not used, because since sample weights are balanced through the total number of pixels, it means that all samples from all patients are already balanced.

#### The complete presentation of the tested combinations

**Fig. 4:**
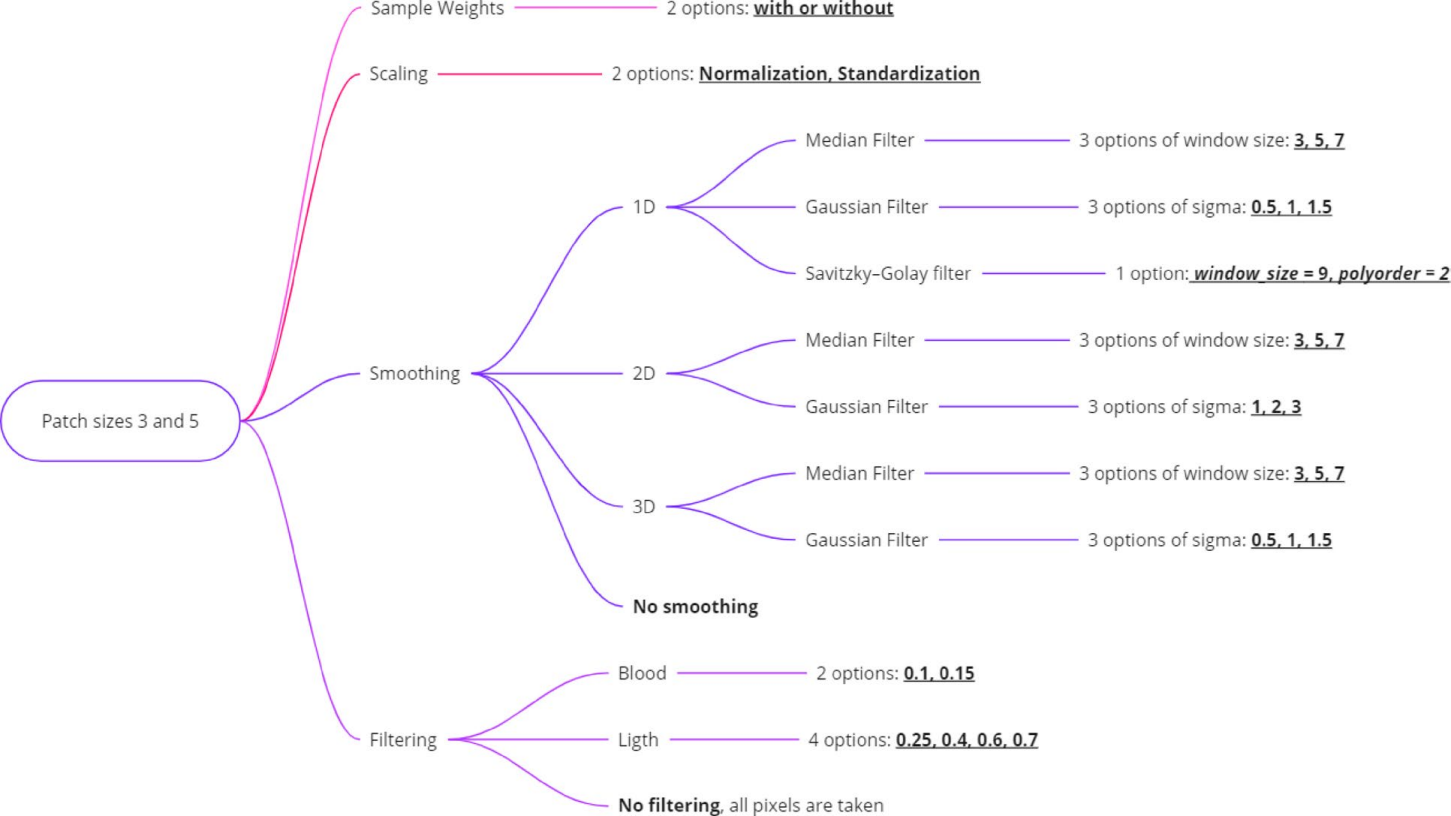
Flowchart depicting various preprocessing options for hyperspectral imaging data, including scaling, smoothing with multiple filters and settings, and filtering based on blood and light characteristics, starting from initial patch sizes of 3 and 5.

In total, 1,584 combinations were tested for both patch sizes 3 and 5 (792 for each patch size). Figure 4 presents the preprocessing options that were permutated, such as Scaling (Normalization and Standardization), Smoothing (1D, 2D, and 3D with Median, Gaussian, and Savitzky-Golay filters), and filtering specific to blood and light characteristics. Smoothing options are parametrized with various window sizes and sigma values. Additionally, Sample Weights can be applied or omitted. This organized approach allows for systematic testing of different combinations to optimize data processing for subsequent analysis.

### Training

#### Inception-based 3D-CNN architecture and cross-validation

**Fig. 5:**
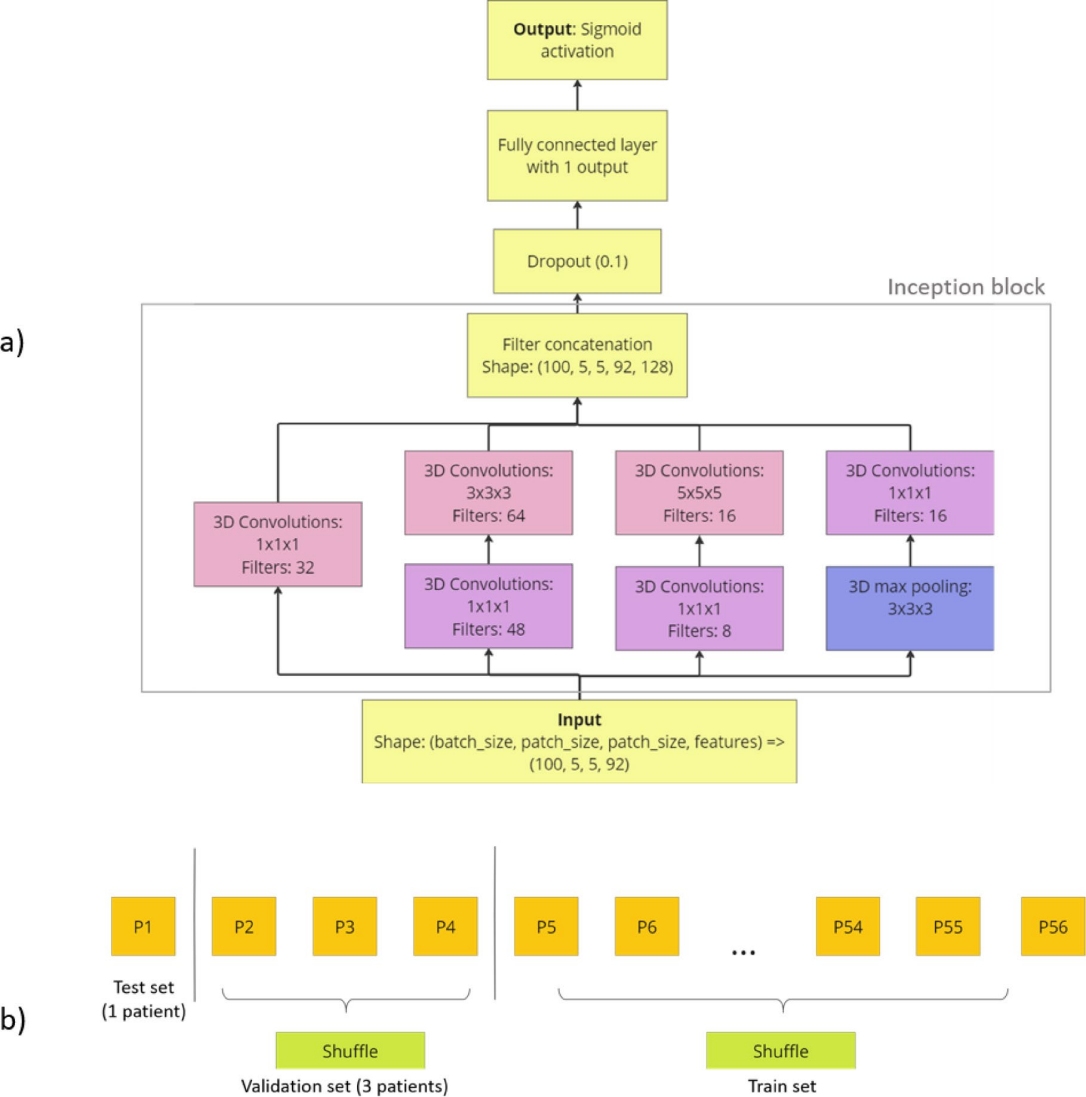
(**а**) Initial 3D-CNN Inception-based architecture. (**b**) The cross-validation used.

Due to the limited dataset size (56 patients only), the decision was made to adopt pixel-wise classification, thereby increasing the number of samples, which is a common practice in medical imaging. This reframed the segmentation problem as an image classification task, for which the Inception model^27^ is particularly effective. That is the reason a 3D-CNN^22^ based on Inception was chosen. The initial architecture is depicted in Fig. 5a and incorporates a single block from the Inception architecture (designated by the gray rectangle). The Inception model offers numerous benefits, notably the concurrent utilization of multiple kernel sizes. This approach not only facilitates the integration of diverse feature maps but also reduces the likelihood of vanishing gradients. The use of ‘same’ padding in all convolutions ensures the preservation of dimensional consistency, facilitating seamless concatenation after each block.

For each combination Leave-One-Out-Cross-Validation was performed. For our dataset of 56 patients, 56 folds were tested for each combination, where during each fold one patient was excluded as test set, three were used as validation set, the rest were used as train set. The described CV can be seen in Fig. 5b. Different numbers of validation patients were tested preliminary (1, 3, 10, 20), and three validation patients gave the best results.

#### Training parameters and infrastructure

Combinations were trained and evaluated with Python (3.7.4), Tensorflow (2.4.0) on the University Leipzig cluster with eight cores of AMD EPYC 32 core processor CPU and 2–8 RXT2080TI GPUs.

Training parameters:

- Maximal epochs – 50.
- Batch size – 500.
- Loss – Binary cross-entropy.
- Optimizer – Adam with β1 = 0.9 and β2 = 0.99.
- Learning rate – 0.0001.
- Activation – ReLU; last layer – Sigmoid.
- Number of wavelengths – 92.

Early stopping was used to avoid overfitting: If the F1-score on the validation dataset did not improve for more than five epochs, training stopped.

Recommendations for implementation:

- At least 16 RAM, possibly less with smaller batch sizes.
- A processor with several cores would be a benefit.
- At least one GPU would be a big benefit.
- It is important to plan some time to implement parallel processing.

In our setup one combination needs 2–3 h to be trained on the small representative dataset. Training on the whole dataset needs 5–6 days, but with parallel processing it was reduced to ~ 1 day.

## Results

### Metrics

In this section, we use the following abbreviations:

- TP (True Positives): Cancerous tissue correctly identified as cancerous.
- TN (True Negatives): Non-malignant tissue correctly identified as non-malignant.
- FP (False Positives): Non-malignant tissue incorrectly identified as cancerous.
- FN (False Negatives): Cancerous tissue incorrectly identified as non-malignant.

Due to the imbalanced nature of the dataset, sensitivity and specificity were chosen as metrics, allowing a more nuanced understanding of model performance. To preserve clinical validity in the performance estimates, each metric is first calculated independently for every patient *p*:

- Sensitivity (also known as Recall), calculated as the ratio of correctly identified positive cases (TP) to the total actual positive cases (TP + FN).

$$sensitivit{y_p}=\frac{{T{P_p}}}{{T{P_p}+F{N_p}}}$$

- Specificity, measured as the proportion of actual negatives (TN) that are correctly identified.

$$specificit{y_p}=\frac{{T{N_p}}}{{T{N_p}+F{P_p}}}$$

Binary classification requires a decision threshold, which partitions predicted probabilities into non-malignant tissue values (below the threshold) and malignant tissue values (above the threshold). As described in^22^ (Sect. 2.5.2), by systematically varying the decision threshold *t* across the interval [0, 1] produces two cohort-level response curves:

- Mean sensitivity:

$$\:sensitivit{y}_{mean}\left(t\right)=\:\frac{1}{N}\:\sum\:_{p=1..N}sensitivit{y}_{p}\:\left(t\right)$$

- Mean specificity:

$$\:{specificity}_{mean}\left(t\right)=\:\frac{1}{N}\:\sum\:_{p=1..N}{specificity}_{p}\:\left(t\right)$$

where *N* is the number of patients. The optimal threshold *T* is defined at the intersection point, where *sensitivity*_*mean*_*(t) = specificity*_*mean*_*(t)*, due to its ability to balance sensitivity and specificity, thus allowing easy model comparison while managing the trade-off between false negatives and false positives.

The intersection point at *T* is what we report throughout the manuscript as the “mean over sensitivity and specificity” (MSS). This procedure provides a patient‑level aggregate metric while preserving equal importance for sensitivity and specificity.

### Statistical analysis

During analysis, we explored what contributors and values affect MSS, which was the dependent variable that we wanted to improve.

Firstly, analysis of variance (ANOVA) was used to determine significance of each preprocessing contributor separately (Sect. 4.3). ANOVA calculates differences in variance among different groups inside the contributor and how significant these differences are.

For smoothing (Sect. 4.4) and filtering (Sect. 4.5), we wanted to examine how each value of each contributor affected the dependent variable compared to the basic “no smoothing” or “no filtering” respectively.

MSS is a single, continuous response variable confined to the 0–1 range. This means that regressions based on labels, such as logistic regression, are not applicable.

Variance-inflation-factor (VIF) diagnostics revealed no multicollinearity among the predictors, and Q-Q plots showed the residuals closely followed a normal distribution, confirming the normality assumption. Consequently, we applied a two-tailed Ordinary Least Squares regression, whose readily interpretable coefficients quantify how each pipeline factor raises or lowers average performance.

In this work we present the next OLS outputs: coefficient, p-value, and confidence interval.

Coefficient shows the estimated change in the dependent variable for a one-unit change in the contributor, assuming all other variables in the model are held constant. Negative coefficients imply that the contributor influences MSS (dependent variable) negatively, and vice versa for positive values.

Confidence interval shows the interval in which 95% of the differences of contributor’s values fall. Represented visually, the confidence interval shows differences in minimums and maximums of compared boxplots.

P-value shows how significant the coefficient and confidence intervals are.

For the statistical models, *n* was 1584 (which corresponds to the total number of combinations) and α = 0,05.

The Supplementary Materials include an analysis of filtering and smoothing interactions, highlighting both the most and least effective combinations. Supplementary Table S1 presents the best significant (*p* < 0.1) interactions between smoothing and filtering, highlighting combinations with the strongest positive coefficients and confidence intervals, whereas Supplementary Table S2 shows the worst interactions where the absence of blood filtering leads to declines. Supplementary Table S3 lists the six highest-scoring preprocessing combinations, all using patch size 5 with Standardization and yielding MSS up to 0.778 and AUC up to 0.9, while Supplementary Table S4 reports the six lowest-scoring combinations with patch size 3 and Normalization, where MSS falls to about 0.465 and AUC to as low as 0.58.

### Scaling, weights and patch sizes

**Fig. 6:**
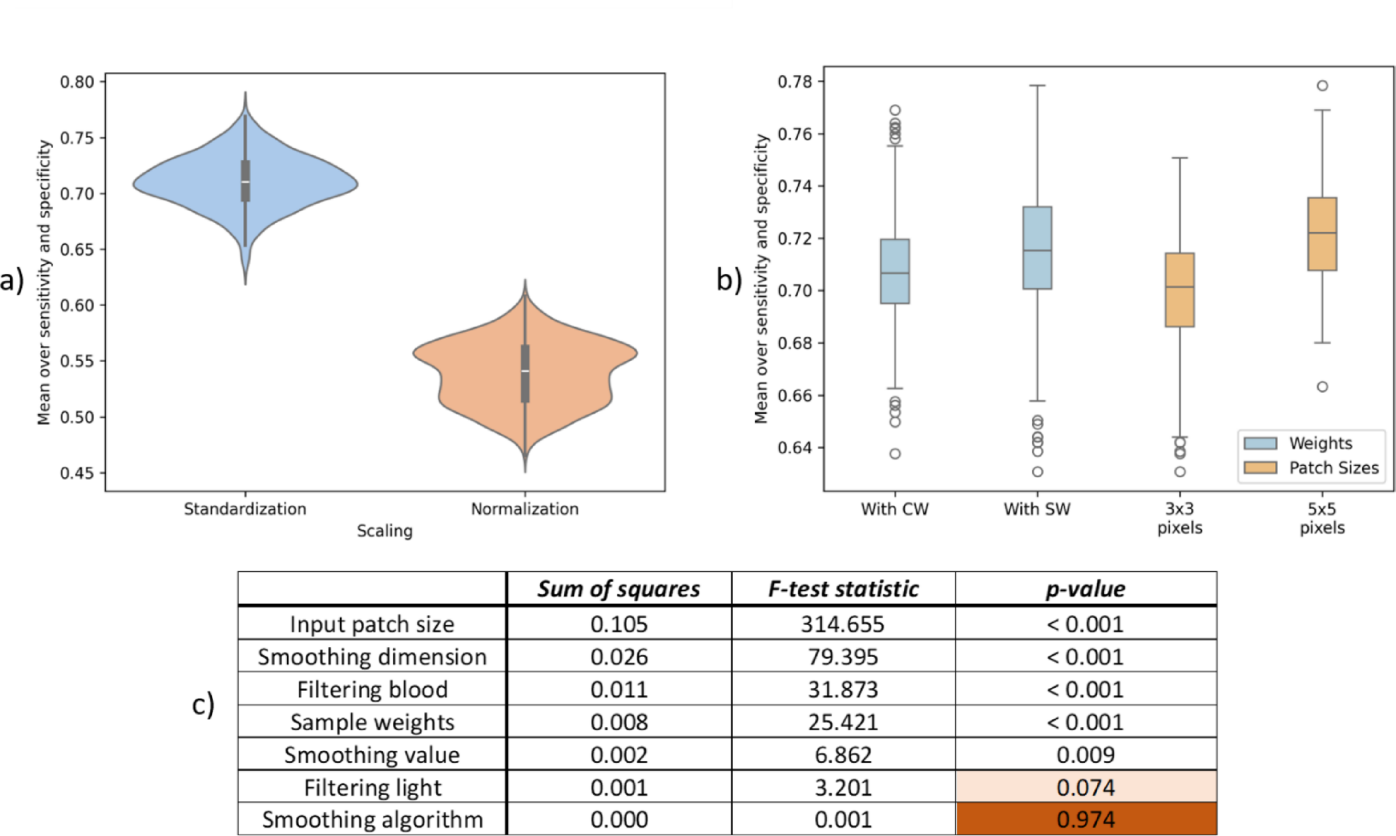
General results. (**a**) Results comparison for two scaling methods, Standardization and Normalization, with Standardization emerging as the most influential pre-processing choice. (**b**) Results comparison for using Sample Weights and Class Weights (blue) and different input patch sizes (orange). Summary: Sample weights outperform class weights, and larger patch sizes lead to better results. (**c**) Results of ANOVA analysis, sorted by “F-test statistic” (from the most to least significant). The not significant contributors are highlighted in red. Summary: Nearly all pre-processing factors proved to be significant, except for the smoothing algorithm and light filtering—their differences have little to no impact on the results.

As Fig. 6a shows, Standardization strongly outperformed Normalization: the median MSS rose from 0.54 with Normalization to 0.71 with Standardization, a mean gain of 17% points. Therefore, only results for Standardization will be analyzed further in the text. The likely explanation is that Normalization compresses spectra into a narrow positive range, which limits variance and obscures subtle tissue differences, whereas Standardization preserves negative values with a wider spread, making feature learning more effective. This question is examined in more detail in the Discussion.

In Fig. 6b the comparison between applying sample weights “SW” and class weight “CW” is shown (blue), as well as differences in input patch sizes (orange). Applying sample weights outperforms applying class weights. By forcing the model to pay more attention to small cancerous areas, sample weights improve sensitivity to rare but clinically critical patterns, helping reduce the risk of missed cancer detections. Although an increased patch size improves results even more (right), since the network could capture more spatial information.

Figure 6c shows the results of ANOVA analysis. The most significant factor (after scaling) is size, which corresponds to visual results from (a) and (b). The second most significant factor is smoothing dimension, which – as will be seen in the next subsection – relies on the fact that 3D smoothing significantly worsens the results. The next are filtering of blood and SW. Notably, filtering of blood is more significant than light filtering.

The ANOVA’s R^2^ value is 0.390, indicating that approximately 39% of the variance in the dependent variable can be explained by the independent variables. Additionally, the p-value for the F-statistics is 7.88 * 10^−80^, demonstrating that the overall model is statistically significant, meaning that the predictors as a group are significantly related to the dependent variable.

### Smoothing

**Fig. 7:**
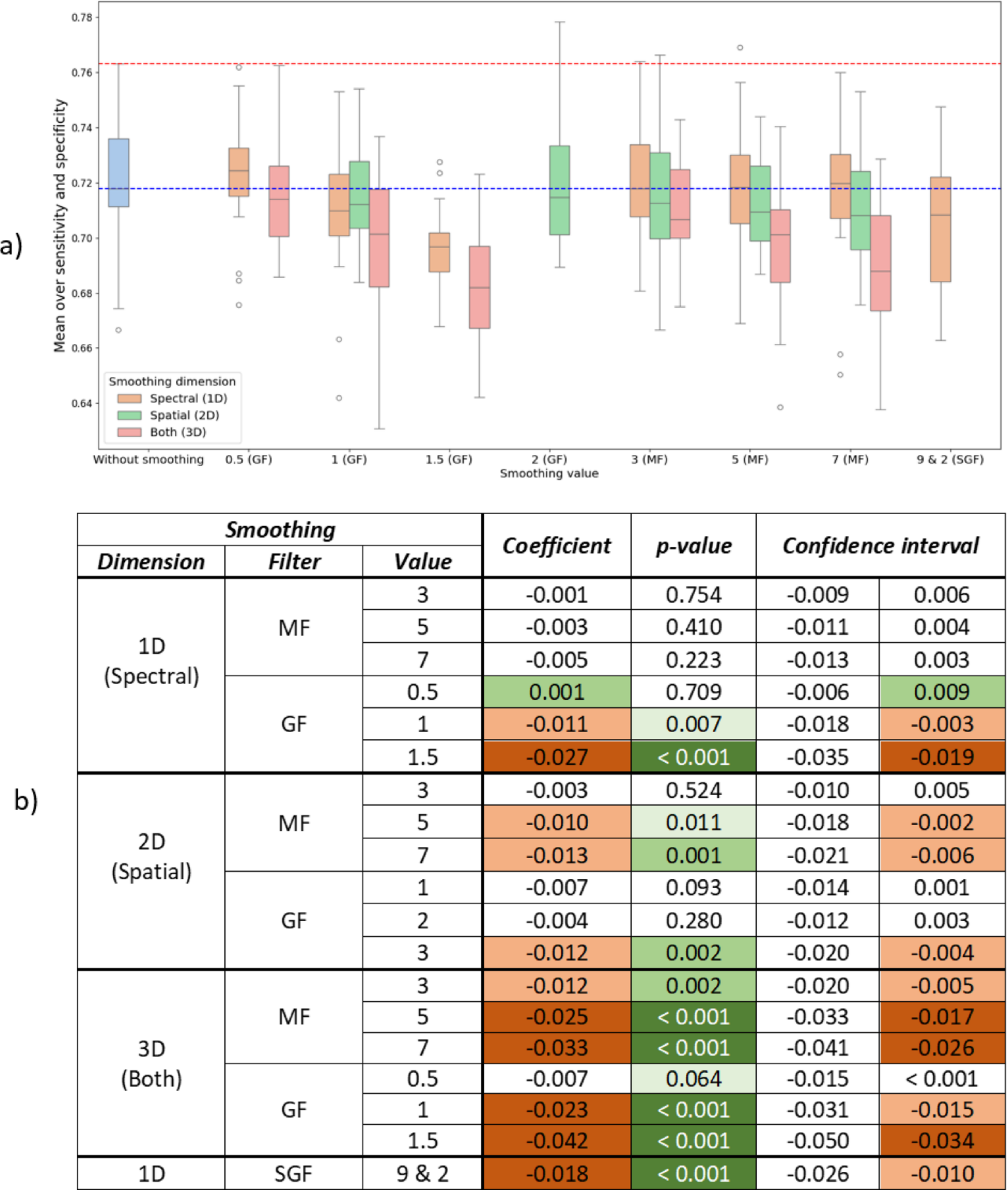
Smoothing results. (**a**) Visual results. The blue boxplot corresponds to absence of smoothing, red dashing line – maximum obtained without smoothing, and blue dashed line – median. X-axis labels are smoothing values with corresponding filters written in parentheses. (**b**) OLS results for smoothing. Shades of green highlight significance, and the only combination that improves sensitivity and specificity (1D, GF 0.5). Shades of red represent severity of bad impact. Standard error (the standard deviation of the estimated Coefficient) for all rows is 0.00393. Summary: 1D, 2D and especially 3D smoothing generally lowers performance. 1D GF 0.5 is the sole configuration that delivers any accuracy gain, although its p-value is > 0.05.

Figure 7a shows the visual results without smoothing applied (blue), and all other tested smoothing combinations. For each smoothing value on the x-axis, the filter is specified in parentheses: GF – Gaussian Filter, MF – Median Filter and SGF – Savitsky-Golay Filter. For each value results for 1D, 2D and 3D (if applicable) are shown. Dashed lines indicate the maximum (red) and median (blue) value achieved with no smoothing applied (blue boxplot). Results of OLS for smoothing are presented in Fig. 7b. R^2^ for the OLS is 0.24 and p-value for F-statistic < 0.0001.

Outcomes:

- Almost all smoothing combinations worsen the results: the medians of all boxplots except for 1D GF (0.5) and 1D MF (7) are below the no-smoothing median (blue line). Although some smoothing combinations could surpass maximal values: 2D GF (2), 1D and 2D MF (3), and with outlier 1D MF (5). These results correspond with the visual results on Fig. 7a and explain why in ANOVA (Fig. 6c) “Smoothing algorithm” is not significant and the “smoothing value” is much less significant than the other contributors.
- Significant results in the Fig. 7b (p-value < 0.05, highlighted in green) correspond to declining outcomes (shades of red), where the highest decline is observed for all 3D smoothing, SGF, and 1D GF 1.5 proving they are not effective.
- Overall Coefficient is negative in all cases in OLS, except for 1D GF 0.5, but these changes are not significant with p-value 0.709. This means that smoothing is worsening mean outcomes. On the other hand, there are combinations, whose 95% interval (the far-right column) is positive, which means that their maximum is higher than maximum without smoothing, but all these changes are not statistically significant (p-value > 0.05). Conclusion would be that if smoothing is used, 1D smoothing with MF 3 or 5 would be the most promising.

### Filtering

**Fig. 8:**
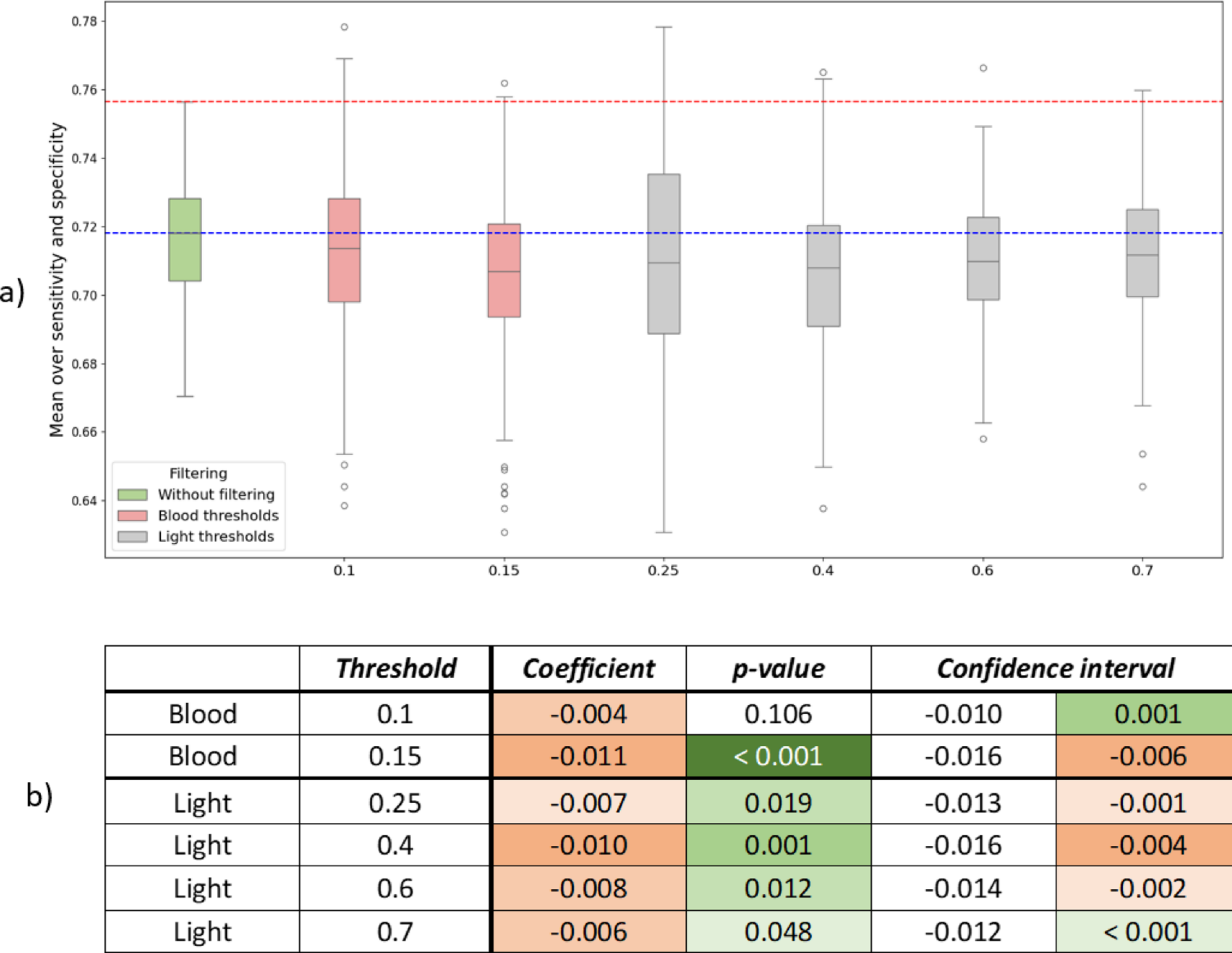
Filtering results (**a**) Visual results: red – blood thresholds, gray – light, green – no filtering. The red dashed line is the maximum obtained without filtering; the blue dashed line denotes the median. (**b**) Results of OLS for blood and light filtering. Shades of green highlight significance. Shades of red represent severity of bad impact. Standard error (the standard deviation of the estimated Coefficient) for all rows is 0.002737. Summary: Filtering is generally counter‑productive; nevertheless, if one chooses to experiment with it, the thresholds that warrant consideration are blood = 0.1 and light = 0.25 or 0.7, with even these settings offering, at best, marginal benefit.

Figure 8a shows the visual results for the different filtering modalities, where green represents no filtering, red – blood thresholds, and gray – light thresholds. The dashed lines represent values without filtering applied: red is the maximum value, blue – the median.

Figure 8b presents the OLS results for blood and light filtering. OLS was calculated separately for blood and light. R2 for the light OLS is 0.015 and p-value for F-statistic equals 0.0197, which means that the changes in light explain the outcomes poorly. R2 for the blood OLS is 0.029 and p-value for F-statistic 0.0001.

The results show:

- Filtering is rather counter-productive, especially light filtering. Filtering worsens median values in all cases (median values of all boxplots are below the blue line), but it could also surpass the maximum value, especially blood threshold 0.1 and light threshold 0.25.
- For blood filtering threshold 0.1 works much better than 0.15 (which significantly worsens the outcomes). But improvements with the threshold of 0.1 are questionable too, because the p-value of improvements is 0.106 (> 0.05).
- According to Fig. 8b, the most promising light thresholds are 0.25 and 0.7, but the improvement is not high.

## Discussion

Based on the ANOVA test and box plot analyses, the factors influencing outcomes can be ranked by importance as follows: (1) Feature scaling; (2) Patch size; (3) Application of sample weights; (4) Blood filtering; (5) Smoothing; (6) Filtering of pixels with reflected light. We will now discuss each of these factors in detail and separately.

The scaling algorithm significantly impacts the outcomes, with Standardization proving to be substantially more effective than Normalization. This can be attributed to the range of values post-scaling. Normalization constrains values to the positive range between 0 and 0.14, resulting in a tighter distribution. Conversely, Standardization yields both positive and negative values, typically ranging from − 3 to 2, facilitating network learning due to the broader value distribution. Although previous research^22^ has indicated that Normalization performed better with Inception-based models, this study suggests otherwise. The discrepancy may be due to differences in cross-validation and the introduction of the Early Stopping technique. Previously, after excluding test patients, the remaining patients were shuffled and split into training and validation sets. In the current study (Sect. 3.3.1), specific patients were excluded as the validation set. Additionally, Early Stopping was employed to prevent overfitting, whereas in the prior study, all networks were trained for the full 40 epochs.

Patch size appears to be an important factor as well, with a tendency that the larger the patch size (i.e., the larger the spatial context given to the model), the better the results. While larger sizes are theoretically advantageous, their practical applications are significantly constrained. For instance, a patch size 7 is already prohibitively demanding in terms of computational resources, including disk space, memory and processing time. Consequently, an alternative approach to consider is the use of super-pixels. Super-pixels can potentially offer a more efficient representation by aggregating pixels into perceptually meaningful regions, thereby reducing computational load while preserving essential spatial information.

Sample weights have a moderate effect. This can be attributed to the fact that annotated cancerous regions are typically smaller than healthy tissue regions. Consequently, the use of sample weights compels the models to give larger attention to samples from patients with fewer annotated regions, thereby forcing the models to learn more from patients with small cancerous areas. This results in improved overall average sensitivity.

The results regarding filtering are inconclusive. On the one hand, the ANOVA analysis on Fig. 6c indicates that light filtering is not a significant contributor. On the other hand, when examining the interaction between filtering and smoothing, light filtering with a threshold of 0.25 demonstrated the biggest potential for performance improvement. Additionally, the results for specific light thresholds are ambiguous, highlighting the necessity to test each threshold individually in each specific case. Regarding blood filtering, a blood threshold of 0.1 is more effective than 0.15. It is important to mention that it is worth exploring additional anomaly-detection techniques—most notably the Reed–Xiaoli (RX)^28^ detector and the Spectral Angle Mapper (SAM)—for the filtering stage. The RX detector flags outliers by exploiting the data’s covariance structure, while SAM measures the angular distance between a candidate spectrum and a reference mean vector. Although both algorithms have proven effective for generic spectral anomaly detection, it remains to be determined whether they can reliably eliminate blood contamination and specular glare. Equally important is deciding how to define the reference mean: should it be derived from the entire dataset, limited to annotated pixels, or estimated separately for each patient. These questions make the line of inquiry particularly promising.

The discussion outcomes that smoothing generally degrades model performance, particularly 3D smoothing, which significantly reduces sensitivity and specificity. This is likely due to excessive smoothing of both spatial and spectral details, which can obscure key features needed for 3D-CNNs to learn useful features. It could also explain why in^14^ SGF improved performance, although it performed poorly in this work. Because in^14^ feed-forward network was used and, in this case, smoothing helps network by filtering of “noise”. But in case of CNNs, this “noise” is useful. This again emphasizes the need of preprocessing tuning separately for each model. Ordinary Least Squares (OLS) regression results confirm that most smoothing techniques, especially those involving 3D operations, correlate with performance declines, although 1D smoothing (along the spectral dimension) shows the least negative impact.

Our findings exhibit varying degrees of translatability to other hyperspectral datasets. Spectral scaling was identified as the most influential preprocessing contributor. Accordingly, when adapting the pipeline to other hyperspectral datasets, scaling—especially Standardization—should be the first parameter evaluated, yet its marked impact necessitates rigorous empirical validation rather than uncritical adoption. Secondly, the benefit of enlarging the input patch size is almost certainly generalizable: in our earlier experiments on several external datasets, increasing the receptive field consistently improved model accuracy, in line with the theoretical expectation that larger image fragments carry richer contextual information and other studies^29^. Second, favoring sample over class weighting is also likely portable, because forcing the network to pay greater attention to rare cancer regions is a broadly applicable principle. In microscopy-style datasets, however—where each hyperspectral cube carries a single label and the annotated area is effectively constant—sample weights lose their meaning, whereas class weights remain pertinent. Third, the utility of spectral smoothing proved algorithm-dependent: for 3D-CNNs it was counter-productive. Yet, it could depend on a hyperspectral camera used. If the camera introduces substantial noise, the potential denoising benefit could outweigh performance loss, so the procedure should be assessed empirically for each camera configuration. Finally, light and blood filtering are the most setup-specific steps; our ex-vivo dataset contains minimal bleeding, whereas in-vivo acquisitions with more blood may show different behavior altogether. In sum, while the key methodological insights on patch size and class weighting appear widely generalizable, the impact of scaling, smoothing and optical-artifact filtering needs to be validated for every new organ and imaging environment.

Future research directions include tuning of the model, including stacking 2 Inception blocks. From the data perspective, the exploration of super-pixels for more efficient image representation is a promising alternative to the currently impractical large patch sizes. Improvements in computational infrastructure will be crucial to support the increased complexity and resource demands of these emerging methodologies. Another way could be to extend the amount of data, using techniques like data augmentation, autoencoders, generative adversarial networks (GANs), zero-shot learning^30–33^.

## Conclusions

This study systematically evaluated 1,584 unique preprocessing configurations, demonstrating that preprocessing has a profound impact on classification performance. The difference between the best and worst-performing combinations exceeded 30% points in MSS (mean over sensitivity and specificity), from 0.46 to 0.77, respectively. However, comprehensively exploring such a large configuration space is infeasible. Therefore, the results emphasize the necessity of developing automated frameworks that can efficiently select representative subsets, tune preprocessing, and ensure strict control over potential confounding factors such as random initialization, data ordering, and leave-one-out cross-validation (LOOCV) consistency. This study provides a comprehensive checklist to guide such efforts.

Several key preprocessing components were identified as particularly influential. Scaling—especially Standardization—substantially improved both sensitivity and specificity and should be considered a default step. Sample weighting, as opposed to class weighting, was more effective in addressing data imbalance, particularly by improving model focus on underrepresented cancerous regions. Additionally, blood filtering showed some slightly positive effects, while filtering of reflected light had limited or negative influence.

Contrary to expectations, smoothing techniques generally degraded performance. Most spatial and spectral smoothing approaches led to performance deterioration, with minor exceptions observed in simple 1D smoothing along the spectral dimension. These results suggest that aggressive noise reduction may remove diagnostically relevant signal features.

Additionally, the classification performance improvement with increasing spatial patch size was observed, consistent with previous literature. Although further enlarging the patch size would be a logical next step, such an approach is computationally impractical. Consequently, future work will focus on segmentation-based strategies. Given the typically small size of medical hyperspectral datasets hindering efficient use of state-of-the-art segmentation models, a promising compromise lies in the use of super-pixels or larger spatial regions for segmentation, rather than relying on pixel-wise patch classification.

In summary, the findings highlight the substantial role of preprocessing in hyperspectral image analysis for cancer detection and outline both methodological guidelines and future research directions aimed at improving model performance while maintaining reproducibility and robustness.

## Supplementary Information

Below is the link to the electronic supplementary material.

Supplementary Material 1

## Data Availability

Data, code and models can be obtained upon request at the following e-mail: marianne.maktabi@medizin.uni-leipzig.de.
