# Supplementary material for: Exploring the role of preprocessing combinations in hyperspectral imaging for deep learning colorectal cancer detection
Source: Sci Rep. 2025 Sep 23;15:32685. doi: 10.1038/s41598-025-20735-x (PMC12457627; doi:10.1038/s41598-025-20735-x)
Supplement: Supplementary file 1 — Supplementary Material 1 [file 41598_2025_20735_MOESM1_ESM.docx]

### Interactions between smoothing and filtering

Since presenting the OLS results of all possible interactions between smoothing and filtering is not practical, we show only the best interactions (Table S1) and the worst interactions (Table S2). For these tables, we used a lower p-value of 0.1 to investigate borderline significant changes as well. R2 for the OLS is 0.35 and p-value for the F-statistic is < 0.0001.

The best interaction in Table S1, highlighted in dark green, is the combination between light filtering with threshold 0.25 and no smoothing: the confidence interval is all positive, and Coefficient and T-test are positive as well (and the highest among all). Another significant combination is 1D MF 3 with light filtering 0.25. A high maximum confidence interval is given by 3D GF 0.5 with light 0.25, as well as no smoothing with light 0.7, although the significance of these improvements is borderline (0.085 and 0.061 respectively).

Notably, among the 4 rows with highest maximal values of confidence intervals and Coefficient, all use light filtering, and two rows use no smoothing.

***Supplementary Table S1.*** *The best significant (p-value < 0.1) interactions of smoothing and filtering. The row in dark green is the best combination. Other green values emphasize improvements in both Coef. and maximal of Confidence interval (the far-right column). L – light filtering, B – blood filtering.*

| ***Smoothing*** | | | ***Filtering*** | ***Coef.*** | ***p-value*** | ***Confidence interval*** | |
| --- | --- | --- | --- | --- | --- | --- | --- |
| ***Dim.*** | ***Filter*** | ***Value*** |  |  |  |  |  |
| 2D | GF | 3 | no | -0.022 ± 0.012 | 0.074 | -0.046 | 0.002 |
| 3D | MF | 5 | no | -0.022 ± 0.012 | 0.069 | -0.046 | 0.002 |
| No | | | L 0.25 | 0.012 ± 0.004 | 0.009 | 0.003 | 0.020 |
| 1D | MF | 3 | L 0.25 | 0.009 ± 0.004 | 0.043 | < 0.001 | 0.017 |
| 3D | GF | 0.5 | L 0.25 | 0.008 ± 0.004 | 0.085 | -0.001 | 0.016 |
| 1D | SGF | 9&2 | L 0.4 | -0.008 ± 0.004 | 0.056 | -0.017 | < 0.001 |
| No | | | L 0.7 | 0.008 ± 0.004 | 0.061 | < 0.001 | 0.017 |
| 1D | GF | 1 | B 0.15 + L 0.25 | -0.015 ± 0.008 | 0.058 | -0.030 | < 0.001 |
| 3D | MF | 7 | B 0.1 + L 0.4 | -0.014 ± 0.008 | 0.084 | -0.029 | 0.002 |

It is also interesting to look at the worst interactions, sorted by Coefficient (Table S2). Notably among the first 5 rows 4 contain “no blood filtering”: blood filtering improves outcomes. For example, no blood filtering makes the already bad 3D GF 1.5 + B 0.15 (the last row) even worse. We can also see among the worst results interactions between blood 0.15 and different light thresholds. It confirms outcomes from Table S1: blood threshold 0.15 worsens outcomes.

***Supplementary Table S2****. The 11 worst significant (p-value < 0.1) interactions of smoothing and filtering sorted by Coefficient, with shades of red present severity of decline in outcomes. L – light filtering, B – blood filtering.*

| ***Contributor 1*** | ***Contributor 2*** | ***Coef.*** | ***p-value*** | ***Confidence interval*** | |
| --- | --- | --- | --- | --- | --- |
| 3D, GF 1.5 | No blood filtering | -0.043 ± 0.012 | < 0.001 | -0.067 | -0.019 |
| 3D, MF 7 | No blood filtering | -0.037 ± 0.012 | 0.002 | -0.061 | -0.013 |
| B 0.15 | L 0.4 | -0.031 ± 0.007 | < 0.001 | -0.044 | -0.019 |
| 3D, GF 1 | No blood filtering | -0.031 ± 0.012 | 0.012 | -0.055 | -0.007 |
| 1D, GF 1.5 | No blood filtering | -0.031 ± 0.012 | 0.012 | -0.055 | -0.007 |
| 3D, GF 1.5 | B 0.1 | -0.024 ± 0.004 | < 0.001 | -0.032 | -0.016 |
| B 0.15 | L 0.7 | -0.023 ± 0.007 | < 0.001 | -0.036 | -0.010 |
| 3D, MF 7 | B 0.1 | -0.020 ± 0.004 | < 0.001 | -0.028 | -0.012 |
| B 0.15 | L 0.6 | -0.018 ± 0.007 | 0.007 | -0.030 | -0.005 |
| B 0.15 | L 0.25 | -0.018 ± 0.007 | 0.007 | -0.030 | -0.005 |
| 3D, GF 1.5 | B 0.15 | -0.017 ± 0.004 | < 0.001 | -0.026 | -0.009 |

### The best and the worst combinations

Supplementary Table S3 consolidates the six highest-scoring preprocessing combinations, each of which employs a patch size of 5 in conjunction with Standardization scaling. These configurations yield mean sensitivity–specificity (MSS) values between 0.763 and 0.778 and achieve areas under the ROC curve (AUC) of 0.87 to 0.91, indicating consistently strong discriminative capacity. In contrast, Supplementary Table S4 presents the six lowest-scoring combinations, all characterized by a smaller patch size of 3 and the use of Normalization scaling. Here, MSS falls to 0.465 – 0.478 and AUC declines to 0.58 – 0.70. Thus, modifying the preprocessing strategy produces an absolute MSS drop of roughly 0.30 (≈ 40 %) and an AUC reduction of about 0.20.

***Supplementary Table S3****. Metrics for the six best preprocessing combinations, sorted by mean over sensitivity and specificity (MSS). SW = sample weights; CW = class weights; MSS = mean between sensitivity and specificity; AUC = area under the ROC curve.*

| **Patch size** | **Scaling** | **Weights** | **Smoothing** | **Filtering** | **MSS** | **AUC** |
| --- | --- | --- | --- | --- | --- | --- |
| 5 | Standardization | SW | 2D, GF 2 | B 0.1, L 0.25 | 0.778 | 0.9 ± 0.15 |
| 5 | Standardization | CW | 1D, MF 5 | B 0.1, L 0.25 | 0.769 | 0.89 ± 0.18 |
| 5 | Standardization | SW | 2D, MF 3 | B 0.1, L 0.6 | 0.766 | 0.91 ± 0.13 |
| 5 | Standardization | SW | 2D, MF 3 | B 0.1, L 0.4 | 0.765 | 0.91 ± 0.15 |
| 5 | Standardization | CW | 1D, MF 3 | B 0.1, L 0.25 | 0.764 | 0.87 ± 0.18 |
| 5 | Standardization | SW | no | B 0.1, L 0.4 | 0.763 | 0.91 ± 0.12 |

***Supplementary Table S4****. Metrics for the six worst preprocessing combinations, sorted by mean over sensitivity and specificity (MSS). SW = sample weights; CW = class weights; MSS = mean between sensitivity and specificity; AUC = area under the ROC curve.*

| **Patch size** | **Scaling** | **Weights** | **Smoothing** | **Filtering** | **MSS** | **AUC** |
| --- | --- | --- | --- | --- | --- | --- |
| 3 | Normalization | SW | 3D, GF 1 | B 0.1, L 0.6 | 0.465 | 0.68 ± 0.27 |
| 3 | Normalization | SW | 2D, GF 3 | B 0.1, L 0.6 | 0.474 | 0.7 ± 0.28 |
| 3 | Normalization | SW | 2D, GF 3 | B 0.15, L 0.6 | 0.476 | 0.7 ± 0.29 |
| 3 | Normalization | SW | 3D, GF 1.5 | B 0.15, L 0.6 | 0.477 | 0.68 ± 0.28 |
| 3 | Normalization | SW | 1D, MF 5 | B 0.15, L 0.4 | 0.477 | 0.58 ± 0.29 |
| 3 | Normalization | SW | 2D, GF 3 | B 0.15, L 0.7 | 0.478 | 0.7 ± 0.28 |
